# A randomised controlled trial to investigate the feasibility and acceptability of a small change approach to prevent weight gain

**DOI:** 10.1007/s10865-023-00455-1

**Published:** 2023-11-06

**Authors:** Henrietta Graham, Claire Madigan, Amanda J. Daley

**Affiliations:** The Centre for Lifestyle Medicine and Behaviour (CLIMB), School of Sport Exercise and Health Sciences, Loughborough University, Loughborough, LE11 3TU UK

**Keywords:** Weight gain prevention, Feasibility and acceptability, Small change approach, Randomised controlled trial

## Abstract

A weight gain prevention strategy showing merit is a small change approach (increase energy expenditure and/or decrease energy intake by 100–200 kcal/day). Studies have tested a small change approach in intensive interventions involving multiple contacts, unsuitable for delivery at scale. The aim here was to assess the feasibility and acceptability of a remote small change weight gain prevention intervention. A randomised controlled trial of 122 participants was conducted. The intervention was a remote 12-week small change weight gain prevention programme (targeting dietary and/or physical activity behaviours). The comparator group received a healthy lifestyle leaflet. Data were collected at baseline and 12-weeks. The primary outcome was the feasibility and acceptability, assessed against three stop–go traffic light criteria: retention, number of participants randomised per month and adherence to a small change approach. Participants’ opinions of a small change approach and weight change were also measured. The traffic light stop–go criteria results were green for recruitment (122 participants recruited in three months) and retention (91%) and red for intervention adherence. Most participants (62%) found a small change approach helpful for weight management and the mean difference in weight was − 1.1 kg (95% CI − 1.7, − 0.4), favouring the intervention group. Excluding intervention adherence, the trial was feasible and acceptable to participants. Despite adherence being lower than expected, participants found a small change approach useful for weight management and gained less weight than comparators. With refinement to increase intervention adherence, progress to an effectiveness trial is warranted.

ISRCTN18309466: 12/04/2022 (retrospectively registered).

**Supplementary Information:**

The online version contains supplementary material available at 10.1007/s10865-023-00455-1.

## Introduction

A large percentage of the global population are living with overweight or obesity (World Health Organization, 2022) and are at increased risk of developing several chronic illnesses including type 2 diabetes, heart disease and cancer (Institute and of Diabetes and Digestive and Kidney Diseases, 2022). Some evidence suggests that adults gain between 0.5 and 1.0 kg each year (Hutfless et al., 2013; Williamson 1993), and this can lead to the development of obesity over time. With limited success in reducing the prevalence of obesity thus far, a better approach may be to focus on preventing weight gain in the first place.

A weight gain prevention strategy that may have merit is a small change approach, which suggests it may be possible to prevent the average 0.5–1 kg of weight gain in the adult population, by decreasing energy intake and/or increasing energy expenditure by 100–200 kilocalories (kcals)/day (Hill, 2009). It is hypothesised that small lifestyle changes will be easier for people to integrate into their everyday life and maintain over time than larger lifestyle changes (e.g., 500 kcal deficit), thus leading to more sustained weight management (Hill, 2009). A small change approach may increase self-efficacy to adhere to weight management which may in turn stimulate additional changes that lead to larger changes over time (Hill, 2009).

Researchers conducted a systematic review (n = 21) and meta-analysis of randomised controlled trials (RCTs) and found adults using a small change approach gained 0.7–0.9 kg less than those who did not over ~ 14 months (Graham et al., 2022). Although the review showed a small change approach may be an effective weight gain prevention strategy in adults, most studies tested the approach within interventions that included multiple support contacts over time. Therefore, it is not clear whether a small change approach as a single strategy message is the effective component, or whether the changes seen in this approach are driven by the multiple contacts within interventions. Furthermore, few studies in the review tested interventions that could easily be delivered at scale within a public health setting.

Therefore, the aim of this study was to examine the feasibility and acceptability of a small change approach intervention for weight gain prevention, developed using the Behaviour Change Wheel (BCW) (Michie et al., 2014) and Self-Regulation Theory (Kanfer & Goldstein, 1975), that is delivered with minimal contact between the research team and participants, and which could be delivered within a public health setting.

## Methods

### Trial design

This was an individual two arm feasibility randomised controlled trial (RCT), with a nested qualitative study (Graham et al., 2022), where participants received either (1) generic information about how to lead a healthy lifestyle (comparator group) or (2) a small change approach intervention (intervention group). All participants were given a £10 Amazon voucher for completing the study. Favourable ethical approval was obtained from Loughborough University (Reference number: 2022-6320-7796). Due to an administrative error the trial was retrospectively registered on the ISRCTN registry (https://doi.org/10.1186/ISRCTN18309466).

### Recruitment of participants

The public were invited to participate in a weight gain prevention study to compare two methods of preventing weight gain, via an advertisement poster shared through social networks, online and paper newspapers and through notice boards in coffee shops and supermarkets. The advertisement poster stated potential participants should scan a QR to read a participant information sheet and to complete an expression of interest form if they wished to take part. Individuals who expressed interest in taking part were contacted by the lead researcher via email and asked to provide their informed consent. Once informed consent was obtained, individuals were contacted once again by the same researcher via email and asked to complete the eligibility screening questionnaire online.

### Eligibility criteria

Participants were eligible if they had a BMI between 20 and 30 kg/m^2^ (healthy weight and overweight), were ≥ 18 years, had access to the internet, owned a smartphone that had a UK telephone number and could speak and understand English.

### Randomisation

An independent statistician produced a computer-generated randomisation list, using STATA (StataCorp, 2021), with a 2:1 randomisation to the intervention and comparator group, stratified by BMI (20–24.9 kg/m^2^ or 25–29.9 kg/m^2^) and using block sizes of six. Two to one randomisation was chosen to maximise the data available for the intervention group, given that this was a feasibility trial. The randomisation list was sent directly from the statistician to a staff member, not otherwise involved in the trial. Each time a participant had provided their consent and completed all the baseline measures, the lead researcher provided the staff member with the participant’s ID number and their BMI category and asked them to randomise the participant. Participants were invited to take part in a weight gain prevention trial that would compare two ways to stop people gaining weight. Participants in both groups received an intervention and were not explicitly told whether they were in the intervention or comparator group and were therefore blinded to study allocation. Due to the nature of the trial, those delivering the intervention were aware of group allocation after assignment.

### Intervention

The intervention was a 12-week behavioural weight gain prevention programme that aimed to encourage participants to implement a small change approach to prevent weight gain by making one small change each day, or seven small changes each week (self-selected or from the list of examples provided), that would decrease their calorie intake and/or increase their energy expenditure by 100–200 kcal. The intervention was developed using the BCW (Michie et al., 2014) and is based on Self-Regulation Theory (Kanfer & Goldstein, 1975), as weight gain prevention requires long-term self-management skills to regulate and adapt behaviour to changing circumstances (Genugten et al., 2010). The intervention was designed to be delivered remotely, via a video and text messages, and to replicate how a small change approach might be implemented within a real-world public health setting (e.g., within Public Health England’s Better Heath campaign). Participants were sent one 10-min animated video (supplementary material 1) via email after they had been randomised to the study. Participants were sent a total of 33 one-way automated text messages (throughout the 12-week intervention), of which 22 were designed to encourage self-regulation by using relevant behaviour change techniques. The behavioural change techniques (Michie et al., 2013) included in the text messages were: goal setting (behaviour), self-monitoring (behaviour), review behavioural goals, discrepancy between current behaviour and goal, problem solving and action planning. As the goal was to develop an intervention that could be delivered at scale, communications with participants were not tailored according to whether self-monitoring occurred, and feedback on behaviour and/or weight was not offered. Six of the text messages were designated to collect data about adherence to a small change approach and five were designed to increase motivation. All 33 text messages are available in Table S1. The components of the interventions are described in Table 1.

**Table 1:** Intervention Components

Intervention component	Description
*Intervention group*	
Animated educational video	A 10-min educational video that explained what a small change approach was and how to use it
Text messages	Text messages that aimed to remind participants of their small change goal and offered support and encouragement to achieve this goal
List of small dietary and physical activity changes	A list of ten small physical activity changes and ten small dietary changes that participants could use to prevent weight gain. (Use of this list was optional)
Small change diary	A diary that participants could use to record the small changes they made over the 12-week intervention period
*Comparator group*	
“Get Healthy” leaflet	A leaflet containing generic information about how to lead a healthy lifestyle

## Outcomes

### Primary outcome

The primary outcome was the feasibility and acceptability of the intervention that used a small change approach to decrease calorie intake and/or increase energy expenditure by 100–200 kcal per day to prevent weight gain. This feasibility study aimed to determine whether progress to a RCT to test the effectiveness of the intervention is justified.

The feasibility of the intervention was assessed for those randomised to the intervention group over the 12-week intervention using the participant retention at follow-up and the number of participants randomised per month (see Table S2). The acceptability of the intervention was assessed over the intervention period using questionnaires to determine the percentage of participants who reported they had made seven small changes over the previous seven days (within bi-weekly questionnaires) and the percentage of participants who found a small change approach and intervention materials helpful/very helpful. Helpfulness was measured using a 5-point Likert scale, ranging from very unhelpful to very helpful.

### Stop–go criteria

The percentage of participants who reported making at least seven small changes to their diet and/or physical activity over the previous seven days, the number of participants randomised per month and retention at follow-up were measured using progression (stop–go) criteria. Including progression criteria in feasibility trials is now common practice, to evaluate whether progression to a larger effectiveness trial is justified (Herbert et al., 2019). The “traffic light” red (do not proceed)/amber (proceed with modifications)/green (proceed) system is a method of representing the progression for a trial in a simple and transparent way and has been used widely in other feasibility studies (Avery et al., 2017). For example, ~ 62% of pilot studies found on the National Institute of Health Research used this approach in 2016 (Herbert et al., 2019). The specific progression criteria for this trial are presented in Table 2.

**Table 2:** Feasibility and acceptability criteria

	Red	Amber	Green
Percentage of participants who reported making at least seven small changes over the previous seven days during week 2, 4, 6, 8, 10 & 12	< 60%	60–79%	≥ 80%
Number of participants randomised per month	Less than 11	Between 12 and 15	At least 16
Retention of participants at follow-up	Follow-up data collected from less than 60% of participants	Follow-up data collected from 60 to 79% of participants	Follow-up data collected from at least 80% of participants

### Secondary outcomes

Secondary outcomes were the mean difference in weight, physical activity behaviours (moderate to vigorous physical activity (MVPA) minutes/week) and the change in the quantity and frequency of ten different foods consumed each week. Although the trial was not powered, measurement of these outcomes were included to assess their completeness by participants as they may be considered as primary outcomes in a subsequent effectiveness trial.

Secondary outcomes were assessed for the intervention and comparator group at baseline and 12 weeks. All outcome data, except weight, were collected using online questionnaires. Weight data was collected through photographs sent via email. Participants were asked to send a photo of themselves standing on weighing scales (camera facing towards their feet) that clearly displayed their weight. Participants were asked to weigh themselves in the morning, after going to the toilet and whilst wearing light or no clothing.

The Exercise Vital Signs Questionnaire (EVSQ) was used to measure participants’ physical activity (Coleman et al., 2012). Participants were asked how many days per week they engaged in moderate to vigorous intensity physical activity (MVPA) (like a brisk walk) and how many minutes they engaged in physical activity at this level. Responses to each of the two items were multiplied together for each participant to calculate their MVPA minutes per week. Initial validation of the EVSQ found good face and discriminant validity and evidence it may provide more conservative estimates of MVPA behaviour when compared to national surveys (Coleman et al., 2012).

As there is no specific measure of dietary behaviour for a small change approach, one was developed for this study to better understand if participants changed their behaviour throughout the intervention period. Participants were asked to indicate the frequency (less than once per week, 1–2× each week, 3–4× each week, 5–6× each week, one per day or two or more times per day) in which they consumed ten different food and drink items (biscuits, hot drinks, cakes, cheese, crisps, sugary drinks, potatoes, side breads, chips and spreads) that were included in the list of examples of small dietary changes. They were also asked to indicate the and portion size (small, medium or large) they typically consumed of these items. Responses to the frequency questions were scored between 0 and 5. Responses to the portion size questions were scored between 1 and 3 (between 1 and 2 for the drink items). Overall scores for dietary behaviour were calculated by adding total frequency and total portion size scores together. The scale had a range of overall scores between 0 and 78, with higher scores indicating a greater consumption (in terms of frequency and portion size) of the items measured.

### Process outcomes

Process outcomes were included to test and understand how the intervention might work to prevent weight gain. Process outcomes examined the change in self-regulation, self-efficacy for dietary behaviours, self-efficacy for physical activity behaviours and cognitive restraint of eating from baseline to follow-up. Process outcomes also examined the small changes that intervention participants made and their opinions towards the intervention materials.

Process outcomes were assessed for the intervention and comparator group at baseline and 12-weeks. Cognitive restraint of eating was assessed using the revised version of The Three Factor Eating Questionnaire (TFEQ-R18) (Karlsson et al., 2000). Participants were asked to read six statements and questions and respond to these using a four-point (questions one to five) and eight-point (question six) Likert scale. Responses to each of the six items were given a score between one and four. For the eight-point question, scores of one and two were then coded as one, scores of three and four were coded as two, scores of five and six were coded as 3 and scores of seven and eight were coded as four. Total scores were calculated by summing the coded scores for each item. Higher scores indicated greater cognitive restraint. The TFEQ-R18 demonstrated good internal consistency with a Cronbach’s coefficient of 0.77 for the six-items related to cognitive restraint (Karlsson et al., 2000).

Self-efficacy for dietary behaviours related to weight management was assessed using the shortened version of the Weight Efficacy Lifestyle Questionnaire (WEL-SF) (Ames et al., 2012). Participants were asked to read eight statements and to use a 11-point Likert scale (0 = not at all confident, 10 = completely confident) to indicate how confident they felt about being able to successfully resist overeating in the presence of negative emotions and in situations with increased food availability and social pressure. Total scores (between 0 and 80) were calculated by summing the responses to each question, with higher scores indicating greater confidence in one’s ability to control eating behaviour. The WEL-SF appears to be a psychometrically valid measure of eating self-efficacy as it accounts for 94% of the variability in the original version (Ames et al., 2012, 2015). Self-efficacy for physical activity behaviours related to weight management was assessed using the Self-Efficacy for Exercise Scale (Rossen & Gruber, 2007). Participants were asked to use a 11-point Likert scale (0 = not at all confident, 10-completely confident) to rate how confident they would be to engage in physical activity in nine different circumstances. Total scores (between 0 and 80) were calculated by summing the responses to each question, with higher scores indicating greater self-efficacy for exercise. Preliminary testing provided evidence for the reliability (Cronbach’s alpha = 0.92) and validity of the ESES (Rossen & Gruber, 2007). Changes in self-regulation behaviours were measured using the shortened version of the Self-Regulation Questionnaire (SSRQ) (Neal & Carey, 2005). Participants were asked to read 31 statements/questions related to their ability to regulate their behaviour to achieve their goals and asked to use a five-point Likert scale (1 = strongly disagree, 2 = disagree, 3 = uncertain or unsure, 4 = agree, 5 = strongly agree) to indicate to what extent they agreed or disagreed with each item. Total scores (from 31 to 155) were calculated by summing the responses to each statement/question, with higher scores indicating greater levels of self-regulation. The SSRQ has good internal consistency (Cronbach’s alpha = 0.92) and correlated strongly with the original 63-item scale (r = 0.96) (Neal & Carey, 2005). A comments box was provided at the end of each bi-weekly questionnaire and some participants provided examples of what small changes they made. These were collated to enrich the understanding of how participants implemented a small change approach throughout the intervention. Following the intervention, semi-structured interviews were held with 18 participants from the intervention group. These findings are reported separately.

Information about the measurement of all outcomes is displayed in Table S2.

### Sample size

As this was a feasibility trial to inform the design of a subsequent effectiveness trial, a formal sample size calculation was not conducted. A sample size of at least 70 participants has been recommended for feasibility trials (Teare et al., 2014). To ensure 70 participants were randomised through 2:1 randomisation to the intervention group and at least 35 were randomised to the comparator group, a sample size of 105 was required. Allowing for 15% drop-out rate, we aimed to recruit 120 participants with 80 randomised to the intervention group and 40 to the comparator group.

### Statistical methods

Data from the questionnaires was analysed by the lead researcher who, due to the nature of the study, was not blinded to study allocation. For the secondary outcomes, baseline differences between the groups (including those who did and did not complete follow-up) were checked by comparing frequency distributions of categorical variables and means of continuous variables. Information on the feasibility and acceptability of the intervention was summarised by presenting percentages in comparison to the stop–go criteria. A sensitivity analysis was conducted with missing data imputed using baseline carried forward to assess the degree by which the results would change if all data was analysed. As there were no differences in results between completers and non-completers, only data for completers were used. Adjusted mean differences between groups and the corresponding 95% confidence intervals (CIs) were estimated from one-way ANCOVA models that included adjustment for baseline values. All estimates of differences between groups are presented with two-sided 95% CIs. Prior to analysis, assumptions for ANCOVA (normality of residuals, homogeneity of variances, and outliers) were tested and met. Post-hoc analysis was completed to determine the percentage of intervention and comparator participants that lost up to 0.5 kg, lost up to 1.0 kg, lost more than 1.0 kg, gained up to 0.5 kg, gained up to 1.0 kg, gained more than 1.0 kg or maintained their baseline weight.

## Results

**Fig. 1:**
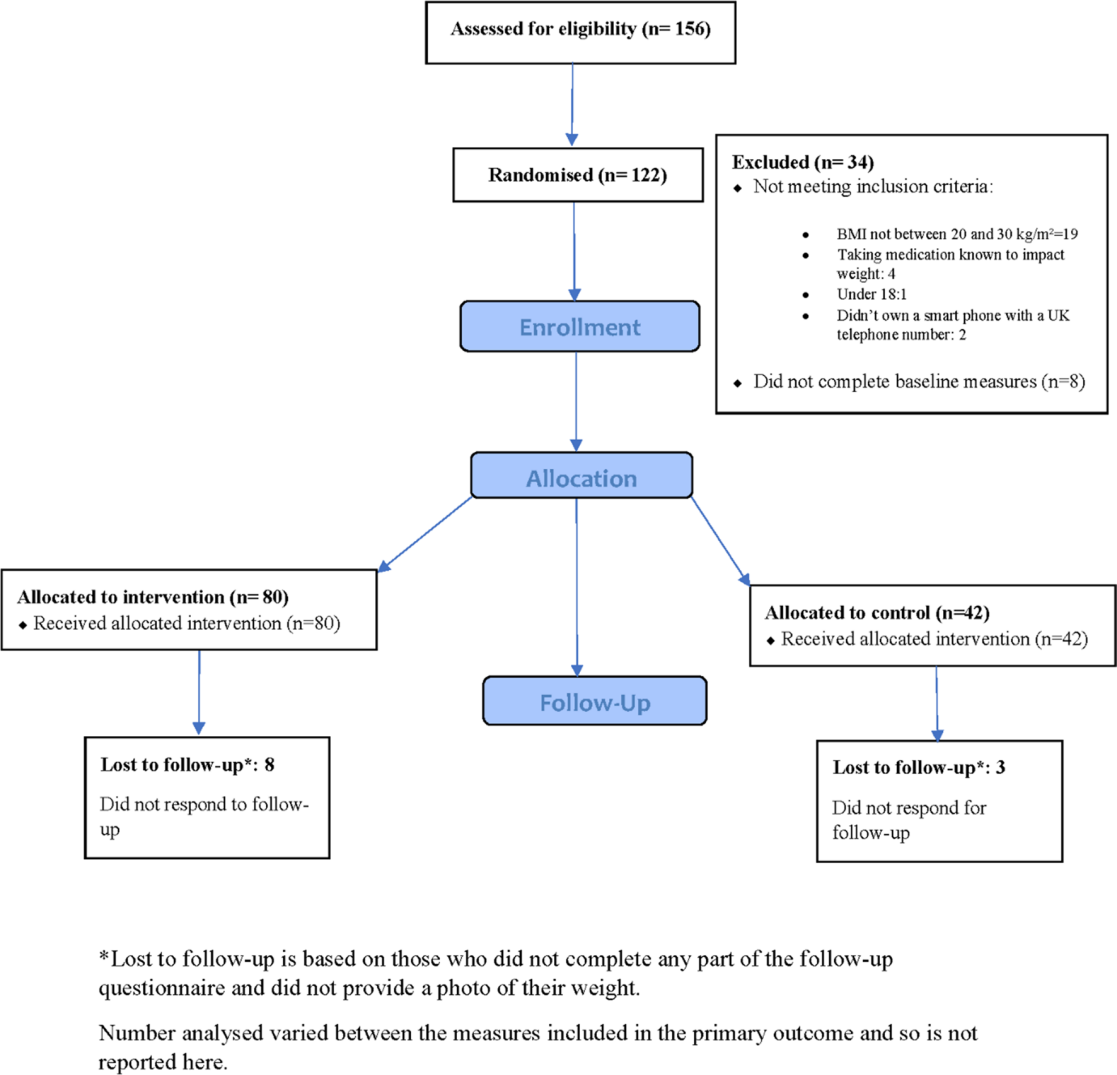
Flow of participants throughout the trial

Participants were recruited between January and April 2022 (11 weeks). The average number of participants recruited each week was 11. In total, 156 participants were assessed for eligibility, of which 34 were excluded (see Fig. 1). One hundred and twenty-two participants were randomised, 80 to the intervention group and 42 to the comparator group. Follow-up took place between April and June 2022 and full data was collected from 111 participants (91%). The trial finished when follow-up data from the last participant enrolled was collected.

### Participants

Most participants identified as female (86.1%) and were of White ethnicity (87.7%). The average age and BMI of participants was 52 years (SD = 13.2) and 25.3 kg/m^2^ (SD = 2.5) respectively. Most participants (66%) self-reported meeting the 150 min/week guideline for participation in MVPA at baseline (World Health Organization, 2021). The groups were generally balanced at baseline for demographic and outcome variables. See Table 3.

**Table 3:** Participant characteristics at baseline

Characteristics	Overall (n = 122)	Intervention (n = 80)	Comparator (n = 42)
Mean (SD) (unless otherwise stated)			
Age, years	52.0 (13.2)	52.3 (13.6)	51.6 (12.4)
BMI, kg/m^2^	25.3 (2.5)	25.3 (2.4)	25.3 (2.6)
Weight, kg	70.0 (10.1)	70.2 (10.4)	69.3 (9.5)
% Female	86.1 (n = 105)	85.0 (n = 68)	88.1% (n = 37)
% White	87.7 (n = 107)	86.3 (n = 69)	90.5 (n = 38)
% Taking medication	41.8 (n = 51)	38.8% (n = 31)	47.6% (n = 20)
Cognitive restraint of eating score	14.9 (3.8)	14.7 (3.9)	15.2 (3.7)
Dietary self-efficacy	36.3 (14.6)	37.5 (15.4)	34.0 (12.7)
Exercise self-efficacy score	45.6 (17.3)	44.7 (17.9)	47.3 (16.1)
Self-regulation score	107.2 (16.7)	106.6 (18.1)	108.3 (13.8)
MVPA minutes/week	233.1 (177.4)	239.4 (190.6)	221.3 (151.4)
Dietary behaviour score	21.0 (8.3)	20.9 (9.0)	21.1 (6.9)

### Primary outcome

The percentage of participants that reported making at least seven small changes within each of the bi-weekly questionnaires ranged from 18 to 30% across the intervention period (Table 3 and Table S3). Sixty-two percent (n = 48/77) of the intervention group reported a small change approach was helpful/very helpful when trying to manage their weight. The percentage of participants who thought the video (59%, n = 45/76), text messages (50%, n = 38/76), list of small dietary changes (63%, n = 48/76) and list of small physical activity changes (63%, n = 48/76) were helpful/very helpful ranged from 50–63%. However, only 41% (n = 31/76) of participants thought the diary, in which they were asked to record how many small changes they made each week, was helpful/very helpful. Approximately 40 participants were recruited each month and as stated above, overall retention was 91%. See Table 4.

**Table 4:** Results for feasibility and acceptability outcomes

Measure	Result						
Acceptability							
Participants who reported making at least 7 small changes within the bi-weekly questionnaire (%)	Week 2	Week 4	Week 6		Week 8	Week 10	Week 12
	28 (n = 21/76)	25 (n = 16/63)	30 (n = 18/60)		22 (n = 12/55)	22 (n = 13/58)	18 (n = 10/55)
Participants who reported a small change approach was helpful/very helpful (%)	62 (n = 48/77)						
Participants who reported the intervention materials were helpful/very helpful (%)	Video	Texts	10 small dietary changes	10 small physical activity changes	Diary		
	59 (n = 45/76)	50 (n = 38/76)	63(n = 48/76)	63 (n = 48/76)	41 (n = 31/76)		
Participants who reported making each type of small change (%)	Diet	Physical activity			Diet + physical activity		
	16 (n = 12/77)	14 (n = 11/77)			70 (n = 54/77)		
Feasibility							
Number of participants randomised per month	40						
Retention (%)	91						

### Secondary outcomes

The mean difference in weight from baseline to follow-up between the intervention and comparator groups was -1.1 kg (95% CI − 1.7 to − 0.4), favouring the intervention group. There was no difference in the dietary behaviour scores (− 1.9, 95% CI − 4.3 to 0.5) or MVPA minutes/week (− 26.1 min, 95% CI − 83.2 to 31.0) between the groups at follow-up. Sensitivity analyses did not alter any of these findings. See Table 5 for results of all secondary outcomes.

**Table 5:** Results for secondary outcomes

Outcome	Baseline	n	Follow-up	n	Mean change within groups*	Mean difference between groups
*Weight (kg)*						
Intervention	70.2 (10.4)	80	69.1 (10.3)	72	− 0.7 (1.6)	− 1.1 (− 1.7 to − 0.4) n = 111
Comparator	69.3 (9.5)	42	69.7 (10.7)	39	0.4 (2.0)	
*Dietary behaviour score*						
Intervention	20.9 (9.0)	80	17.5 (9.3)	75	− 3.3 (7.0)	− 1.9 (− 4.3 to 0.5) n = 115
Comparator	20.8 (7.1)	42	19.4 (7.4)	40	− 1.5 (6.1)	
*MVPA minutes/week*						
Intervention	239.4 (190.6)	80	234.9 (170.8)	72	− 1.9 (163.4)	− 26.1 (− 83.2 to 31.0) n = 111
Comparator	238.7 (183.8)	42	256.1 (202.8)	39	27.8 (151.2)	

*Due to the difference in the number of people within each group analysed at baseline and follow-up, in some cases, the mean change within groups does not match with means presented at baseline and follow-up

### Process outcomes

The mean difference in cognitive restraint of eating (1.6, 95% CI 0.5 to 2.8) and self-regulation (5.0, 95% CI 0.8 to 9.3) scores favoured the intervention group at follow-up. There was no difference in self-efficacy for dietary behaviour (− 0.2 95% CI − 4.8 to 4.3) or self-efficacy for physical activity behaviour scores (− 0.0, 95% CI − 5.3, 5.3) between the groups at follow-up. Sensitivity analyses did not alter any of these findings. See Table 6 for the results of the process outcome analysis. Within the bi-weekly questionnaires, 33 intervention participants provided 63 examples of how they implemented a small change approach to prevent weight gain over the 12-week study. Increasing physical activity was listed as the most common technique used to implement a small change approach. See Table S4 for further details.

**Table 6:** Results for process outcomes

Outcome	Baseline	n	Follow- up	n	Mean change within groups	Mean difference between groups (95% CI) n
*Cognitive restraint of eating score*						
Intervention	14.7 (3.9)	80	17.4 (3.5)	76	2.8 (3.6)	1.6 (0.5 to 2.8) n = 116
Comparator	15.2 (3.7)	40	16.1 (3.7)	40	0.8 (3.5)	
Self-efficacy for dietary behaviours score*						
Intervention	37.5 (15.4)	80	38.6 (15.0)	76	0.8 (13.0)	− 1.0 (− 5.2 to 3.2) n = 116
Comparator	34.0 (12.7)	40	37.3 (14.8)	40	2.9 (8.6)	
*Self-efficacy for physical activity behaviours score*						
Intervention	44.7 (17.9)	80	48.8 (19.5)	76	4.0 (16.2)	− 0.0 (− 5.3 to 5.3) n = 116
Comparator	47.3 (16.1)	40	50.7 (14.7)	40	3.1 (11.7)	
*Self-regulation score*						
Intervention	106.6 (18.1)	80	110.8 (19.0)	75	3.3 (10.8)	5.0 (0.8 to 9.3) N = 115
Comparator	108.3 (13.8)	40	107.3 (16.6)	40	− 2.0 (11.8)	

^*^Due to error, only 7 domains (instead of 8) were measured for self-efficacy for dietary behaviour at baseline. Although the full 8 domains were measured at follow-up, mean scores at follow-up, mean change within groups and mean difference between groups were calculated using the 7 domains only. The mean scores for each of the 8 domains of self-efficacy for dietary behaviour measured at follow-up are reported below

Intervention: domain 1 = 5.7 (3.1), domain 2 = 5.2 (2.6), domain 3 = 5.4 (2.9), domain 4 = 6.7 (2.7), domain 5 = 4.9 (3.2), domain 6 = 4.82 (2.8), domain 7 = 5.9 (3.0), domain 8 = 6.5 (2.8)

Comparator: domain 1 = 5.7 (3.4), domain 2 = 4.5 (2.2), domain 3 = 5.3 (2.9), domain 4 = 6.3 (2.6), domain 5 = 5.0 (3.0), domain 6 = 4.6 (2.2), domain 7 = 5.8 (2.6), domain 8 = 5.5 (2.5)

## Discussion

This study examined the feasibility and acceptability of an intervention that aimed to encourage participants to implement a small change approach to their dietary and physical activity behaviours to prevent weight gain. The recruitment and retention targets were met. The percentage of participants making at least seven small changes to their diet and/or physical activity behaviours each week during the intervention was less than 60%, meaning targets for adherence to a small change approach were not met for this progression criteria. Despite this, most intervention participants (62%) reported they found a small change approach helpful/very helpful when trying to manage their weight and the intervention group gained less weight than comparators at follow-up. Therefore, it may still be possible that a small change approach, delivered as a real-world public health intervention, could be effective in preventing weight gain even when participants make less than seven small changes per week.

The small change intervention developed for this study was based on, and used, behavioural change techniques related to self-regulation theory, as successful weight gain prevention will largely depend on an individual’s ability to regulate and adapt their behaviour to changing circumstances (Graham et al., 2022). Intervention participants’ self-regulation scores increased more than those in the comparator group, indicating that the intervention appears to have worked as intended. This finding is important given that research identifying mediators of successful outcomes in weight management interventions found that self-regulation skills emerged as the most consistent predictor of both short- and long-term weight control in adults (Teixeira et al., 2015).

Intervention participants’ cognitive restraint of eating scores also increased slightly more than those in the comparator group, a finding demonstrated in other weight gain prevention interventions (Mason et al., 2018; Medina, 2016). The role of cognitive restraint of eating in weight management is disputed within the literature, with some research suggesting it can be a facilitator to successful weight management (Phelan et al., 2009) and other research suggesting it can be a barrier to successful weight management (Epstein et al., 2003; Fairburn, 2008; Stice, 2001). However, a recent review that sought to integrate this divergent literature concluded that cognitive restraint of eating cannot be categorised as entirely healthy or unhealthy, but rather it could be health promoting or detrimental depending on the circumstances in which it is employed (Phelan et al., 2009). The role that cognitive restraint plays in a small change approach is therefore undetermined and future research examining a small change approach should seek to understand whether it may be a facilitator or barrier to successful weight management.

This feasibility trial found no difference in the dietary and physical activity outcomes across the groups at 12-week follow-up. The lack of difference in scores between groups for these outcomes may be because this study is a feasibility trial and not statistically powered to detect differences. Additionally, there may have been no changes in physical activity between the groups because most people self-reported meeting physical activity guidelines at baseline (World Health Organization, 2021). Weight was measured objectively, whereas diet and physical activity behaviours were assessed using self-report measures, which may have not captured true changes in behaviour.

As this is the first study to examine the feasibility and acceptability of a small change approach that could be delivered in a real-world public health weight gain prevention intervention, it is not possible to make direct comparisons with other studies. However, the secondary outcome findings of this study are consistent with a systematic review, where adults who used a small change approach to manage their weight gained 0.7–0.9 kg less than those who did not (Graham et al., 2022). Results are also similar to those of another systematic review that investigated the effectiveness of RCTs that aimed to prevent weight gain in adults, where the intervention groups gained 1.2 kg (95% CI -1.50 to -0.80) less than comparators (Martin et al., 2021). This suggests that the small change weight gain prevention intervention used in this study may have the potential to be effective, although the short- and longer-term effectiveness of the intervention needs to be tested.

The difference in weight of 1 kg between the groups at follow up is similar to the result of another study where participants made between 9 and 13 small changes to their indulgence intake each week (Madigan et al., 2018). Whilst a modest difference in weight was recorded between the groups at follow-up in the current study, this should be considered in light of research showing that the average adult gains 0.5–1.0 kg each year (Hutfless et al., 2013; Williamson 1993), which can lead to the development of overweight and obesity over time and that even small changes in weight can have a significant impact. For example, in a modelling study of the Australian population it was found that a reduction of only 0.21 kg (95% uncertainty interval (UI) 0.16–0.25) translated into overall healthcare cost savings of AUD 793.4 million (95% UI 589.1–976.1), or £420 million, over the lifetime of the individuals included in the analysis (Lal et al., 2020). The small change approach intervention delivered within this study may have the potential to offset weight gain and could contribute towards stabilising obesity rates within the population and increase healthcare cost savings over time.

### Strengths and limitations

The study included a process evaluation to investigate how the intervention may work to prevent weight gain, the findings of which may be valuable to intervention developers who wish to use a small change approach to help prevent weight gain in the population. The retention rate was high, with 91% of participants completing follow-up. Despite the study being delivered remotely, objective measures of weight were obtained through asking participants to report their weight via photographs taken on their home scales. This method of data collection reduced the risk of self-report bias whilst also allowing participants to be recruited across a wide geographic area at a time with heightened uncertainty due to COVID-19. This method of collecting weight data has been recommended when objective measurement is not possible (Krukowski & Ross, 2020).

Although attempts were made to recruit a diverse sample by targeting ethnic minority and male Facebook and community groups, the sample was predominately made up of females of White ethnicity. It is therefore possible that the intervention may work differently in more diverse populations. Future research should explore recruitment channels to ensure a more diverse sample is achieved to increase the representativeness of the data collected. The method of using bi-weekly questionnaires to assess adherence to a small change approach was novel. Future research should therefore consider whether the method used here to assess adherence to a small change approach is able to fully capture changes in behaviour and whether additional and/or other methods are needed. Additionally, the analysis used to calculate adherence to a small change approach was based on data from only those who completed the bi-weekly questionnaires (completer analysis). Findings related to adherence therefore represent a best-case scenario and so should be interpreted with this in mind. The dietary behaviour measure developed specifically for this study did not reflect the wider eating behaviour of the participants, rather it focused on assessing changes in dietary behaviour related to portion size and frequency of the consumption of ten different example food and drink items targeted within the intervention. Therefore, a more comprehensive measure of dietary behaviour should be considered for use in future research. Finally, dietary and physical activity behaviour were assessed using self-report measures, the trial was not statistically powered to detect differences in these outcomes and findings may be subject to social desirability or recall (overreporting) bias and consequently results should be interpreted with caution.

### Future research

Results from the primary outcome analysis show that some intervention components were viewed more positively than others. For example, participants reported they found the list of small dietary and physical activity changes more helpful than the educational animated video, text messages and self-monitoring diary. To improve adherence to a small change approach in future research it may be useful to refine the intervention components that were rated as less helpful by participants. It may be advantageous to ask members of the public to give detailed feedback on the animated video to help refine its content, focus and acceptability further. Feedback from the public could also be used to help determine the most appropriate timing and frequency of sending text messages, as these were important factors that influenced satisfaction with text-messages in another weight management intervention (Mcgirr et al., 2020). The text messages could also be used to provide participants with algorithm-based feedback about their weight goal, as feedback is important for supporting behaviour change (National Institute for Health and Care Excellence, 2014). Alternative methods to self-monitor small change behaviour (s) (e.g., smartphones and/or smart watches) should be explored, as the small change diary was rated as the least helpful intervention component. Digital self-monitoring tools could also aid in greater adherence than paper diaries whilst reducing the likelihood of recall bias and improving adherence. Finally, objective measures of physical activity and alternative self-report measures of diet should be considered for use in future research.

## Conclusions

Preventing weight gain in the population is a public health priority. This study provides data to indicate that a small change approach is a feasible and acceptable strategy to help the public prevent weight gain, and it is a strategy that could be effective when delivered at scale within a real-world public health intervention. The results of this study have merit in helping to refine future research on the question of the usefulness of a small change approach for weight gain prevention but an adequately statistically powered trial with longer follow-up is now required.

### Supplementary Information

Below is the link to the electronic supplementary material.

Supplementary file1 (DOCX 2996 kb)
