# Supplementary material for: A randomised controlled trial to investigate the feasibility and acceptability of a small change approach to prevent weight gain
Source: J Behav Med. 2023 Nov 6;47(2):232–43. doi: 10.1007/s10865-023-00455-1 (PMC10944418; doi:10.1007/s10865-023-00455-1)
Supplement: Supplementary file 1 — Supplementary file1 (DOCX 2996 kb) [file 10865_2023_455_MOESM1_ESM.docx]

**The small change study to investigate the feasibility and acceptability of a small change approach to prevent weight gain**

Henrietta Graham^1^, Dr Claire Madigan^1^, Professor Amanda J. Daley^1^

^1^The Centre for Lifestyle Medicine and Behaviour (CLIMB), School of Sport Exercise and Health Sciences, Loughborough University

**Key words**

Weight gain prevention, feasibility and acceptability, small change approach, randomised controlled trial

**Running title**

The small change study to investigate the feasibility and acceptability of a small change approach to prevent weight gain

**Acknowledgments**

AD is supported by a National Institute for Health Research (NIHR) Research Professorship award. This research was supported by the NIHR Leicester Biomedical Research Centre. The views expressed are those of the author(s) and not necessarily those of the NHS, the NIHR or the Department of Health and Social Care.

**Corresponding author**

Henrietta Graham

The Centre for Lifestyle Medicine and Behaviour (CLiMB) Loughborough University

Epinal Way Loughborough LE11 3TU

United Kingdom [h.graham@lboro.ac.uk@lboro.ac.uk](about:blank)

**Competing interests**

The authors declare no competing financial interests.

**Supplementary material 1.** Animated video link

[https://vimeo.com/666029930/81d2bb66c6](about:blank)

**Supplementary Material 2.** Description of how dietary behaviour was measured

Participants were asked to indicate the frequency (less than once per week, 1-2 times each week, 3-4 times each week, 5-6 times each week, one per day or two or more times per day) and portion size (small, medium or large) in which they consumed ten different food and drink items (biscuits, hot drinks, cakes, cheese, crisps, sugary drinks, potatoes, side breads, chips & spreads). Most of the items were discretionary foods/drinks as it was thought that small changes to intake of discretionary foods would be an easy way for participants to reduce their calorie intake by 100-200 kcal/day. However, some non-discretionary foods were included in the list (for example, potatoes) to accommodate those participants who do not consume discretionary foods. See Figure S1 & S2 for an example of a question within the dietary behaviour measure.

Responses to each of the frequency questions were given a score between 0 (less than once per week) and 5 (two or more times per day). Responses to each of the portion size questions regarding the food items were given a score between 1 (equivalent to the small portion size depicted) and 3 (equivalent to the large portion size depicted). Responses to each of the portion size questions regarding the drink items were given a score between 1 (equivalent to the small portion size depicted) and 2 (equivalent to the large portion size depicted). Total frequency scores were calculated by summing the responses to each of the frequency questions. Total portion size scores were calculated by summing the responses to each of the

portion size questions. Overall scores for dietary behaviour were calculated by adding total frequency and total portion size scores together. The scale had a range of overall scores between 0 and 78, with higher scores indicating a greater consumption (in terms of frequency and portion size) of the items measured.

A decrease in score of 2 on the dietary behaviour measure from baseline to follow-up could indicate a decrease in the portion size of crisps from 144g to 30g or a decrease in the frequency of consumption of crisps from 5-6 times each week to 1-2 times each week. A decrease in score of 2 could also indicate a decrease in portion size from 144g to 60g and a decrease in the frequency of consumption from 5-6 times each week to 3-4 times each week.


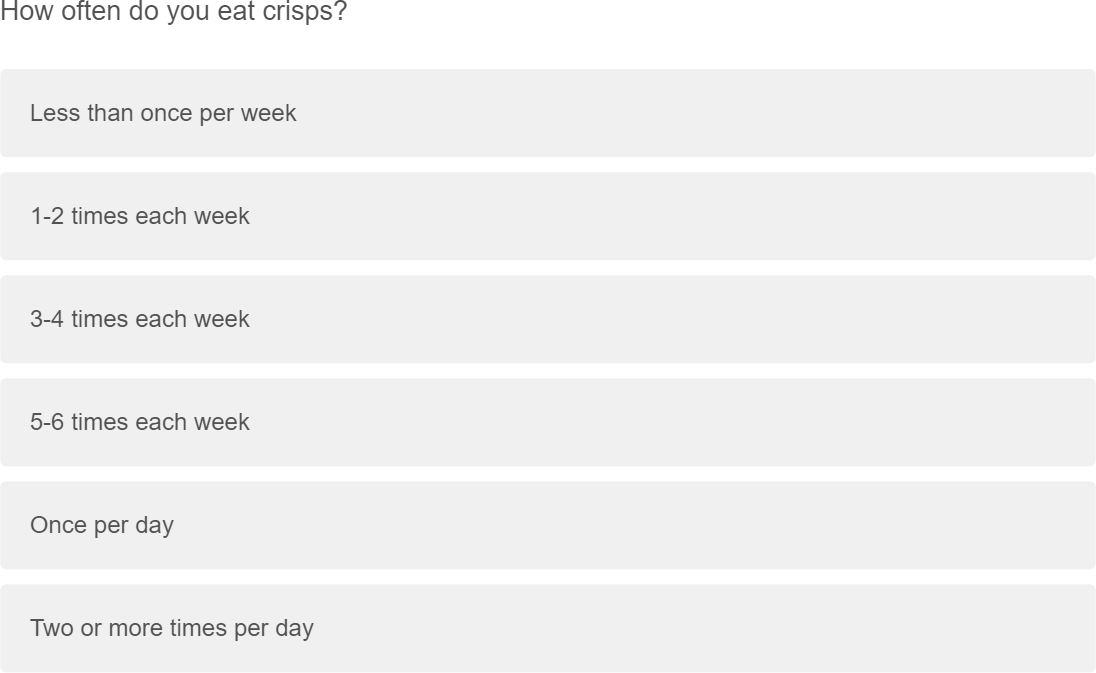


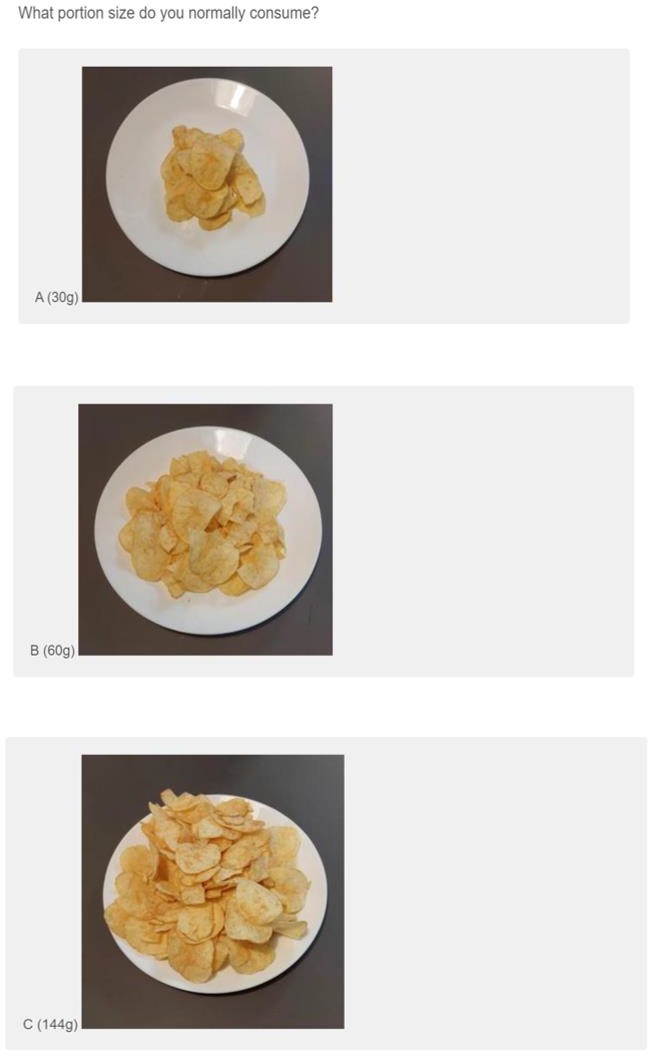


**Supplementary Material 3.** Intervention Development

The Behaviour Change Wheel (BCW) was applied to develop the small change intervention used in this feasibility trial. The process by which this intervention was developed is shown below.


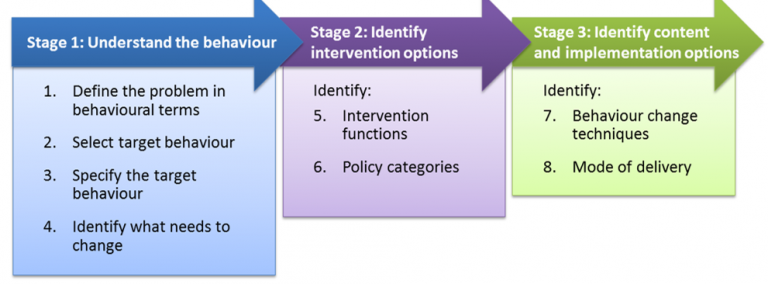


After the BCW was applied, it was determined that the small change approach weight gain prevention intervention would consist of the following:

1. Aim: increase participants psychological capability, reflective motivation, automatic motivation and social opportunity to implement a small change approach to prevent weight gain
2. Intervention functions: education and training about a small change approach and modelling of how to implement it
3. Policy functions: communication and marketing to prompt people to take part in a small change approach intervention
4. BCTs: information about health consequences, prompts/cues, self-monitoring of behaviour, demonstration of behaviour, instruction on how to perform the behaviour. Additional BCTs were chosen to increase self-regulation behaviours. There were: goal setting, review of behaviour goal, discrepancy between current behaviour and goal, problem solving and action planning
5. Modes of delivery: mobile phone (text) & internet (video)

A logic model, underpinned by self-regulation theory, was developed to explain how the small change intervention might lead to weight gain prevention.


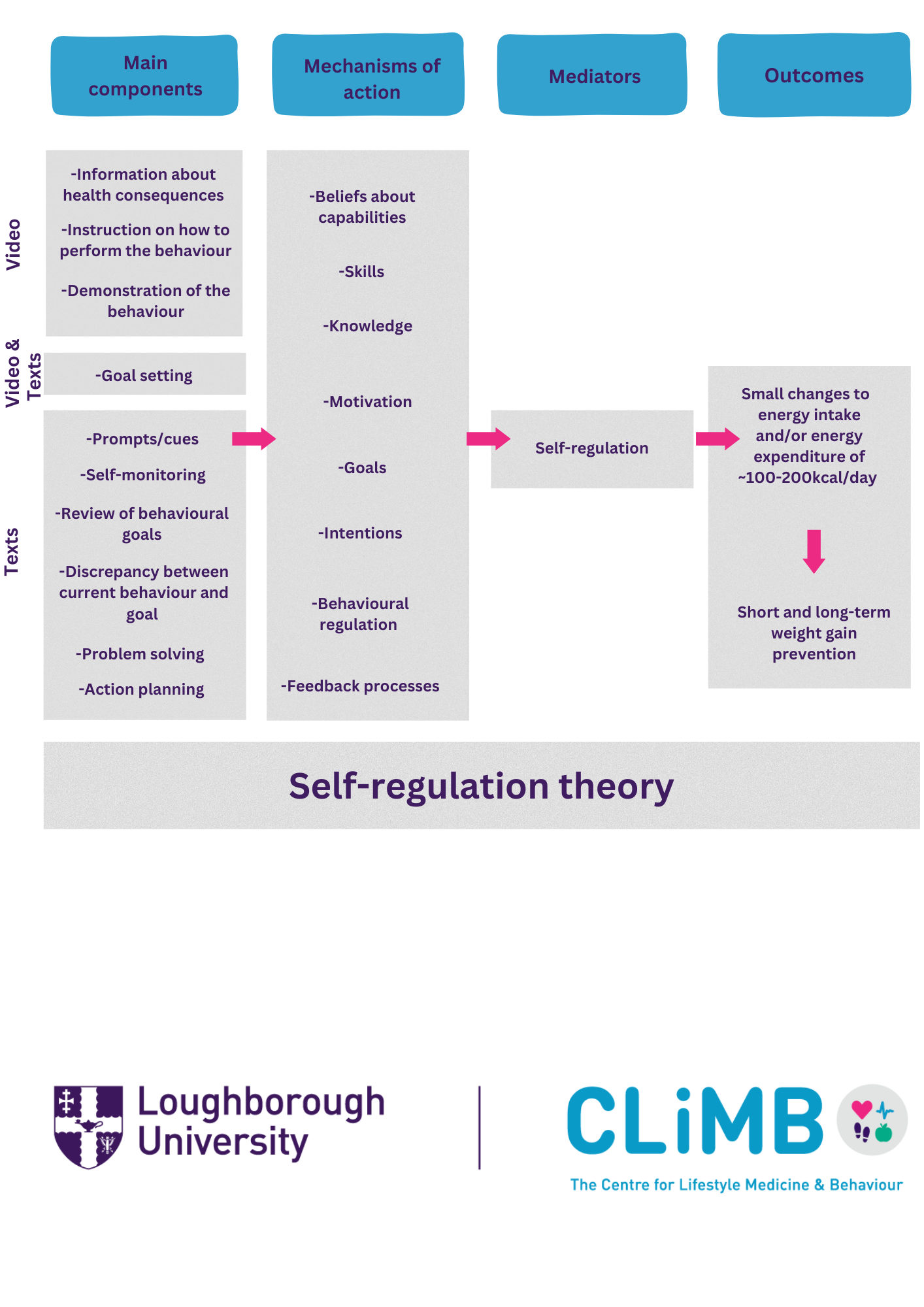


**Supplementary Material 4.** List of small changes provided to participants


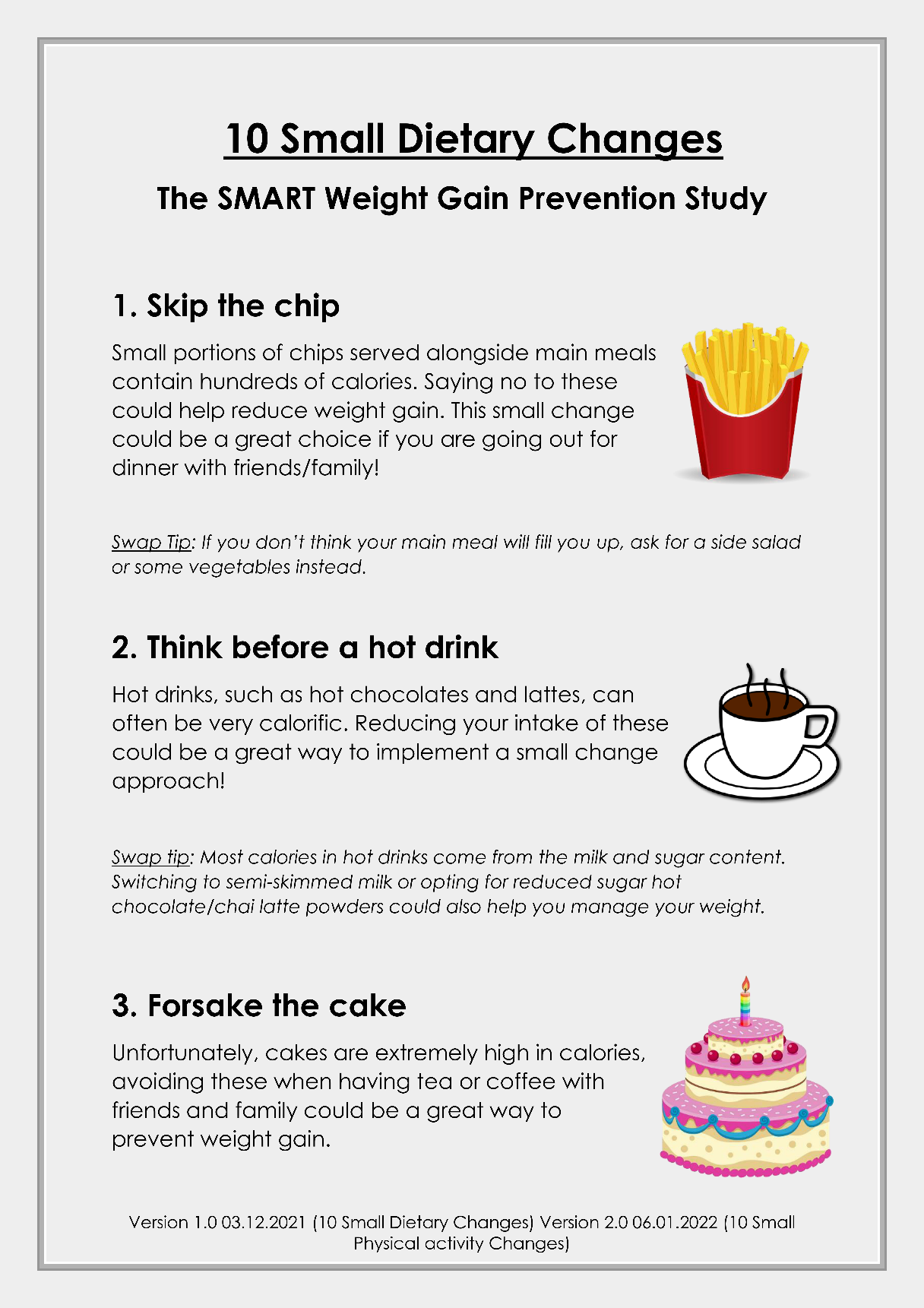


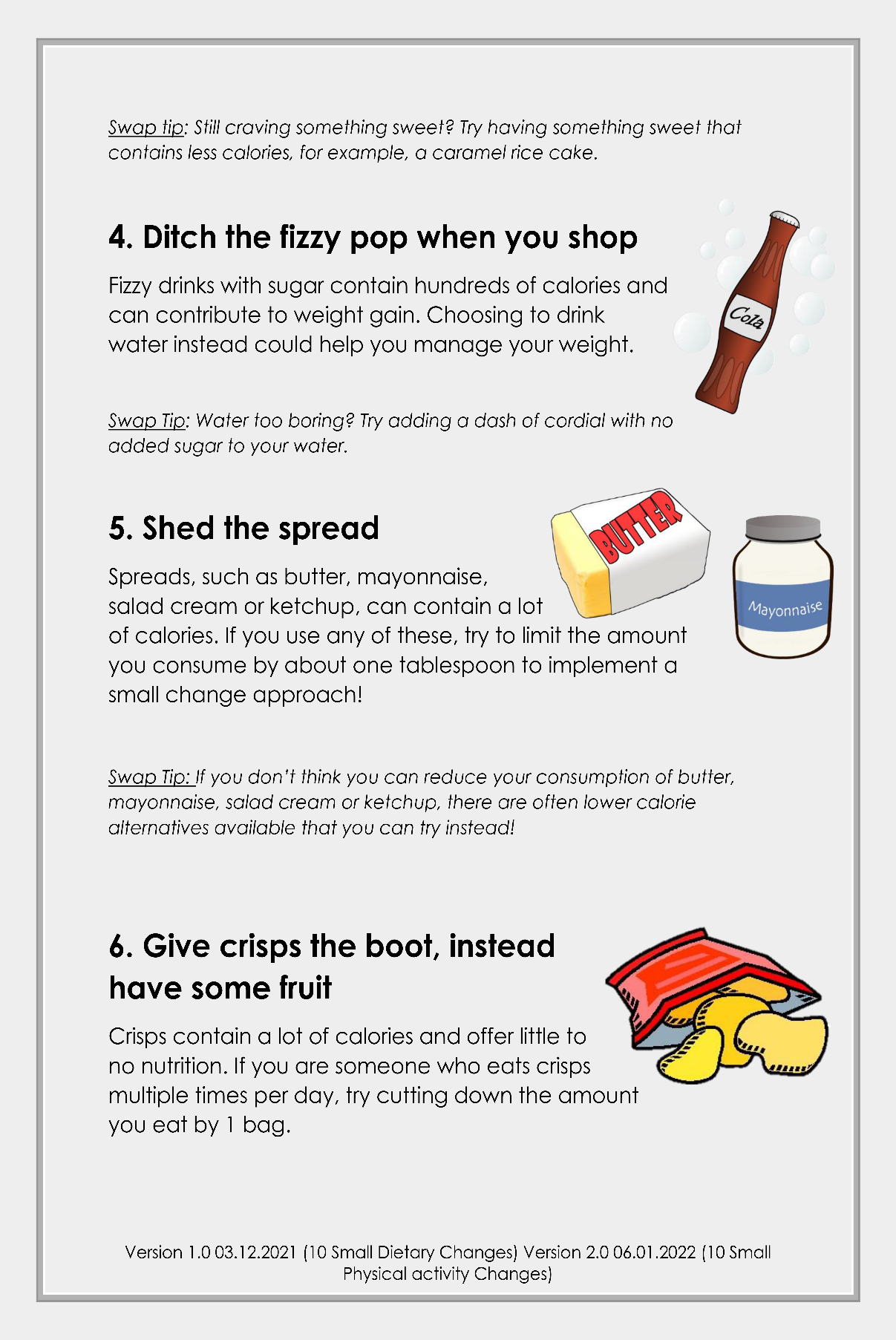


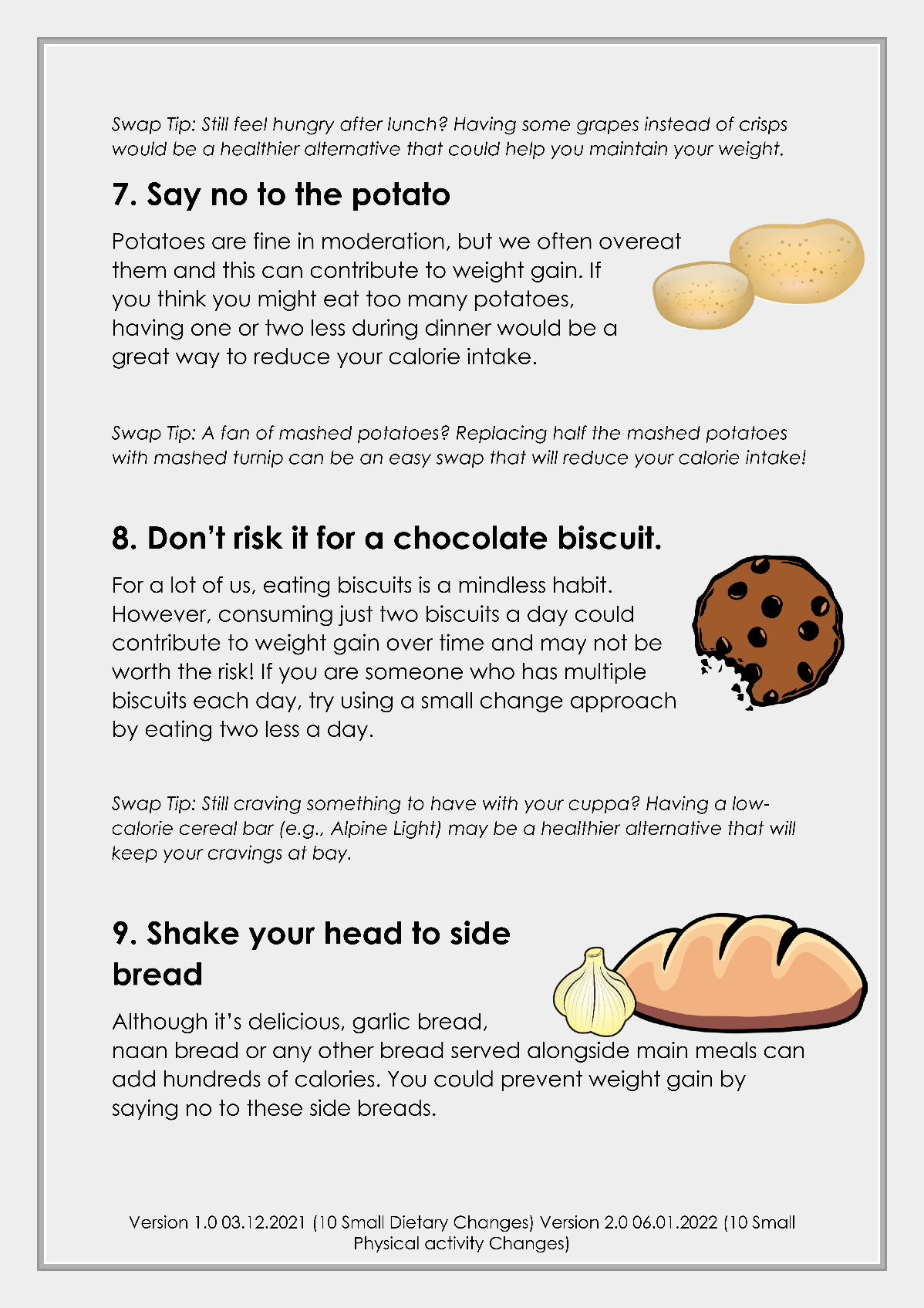


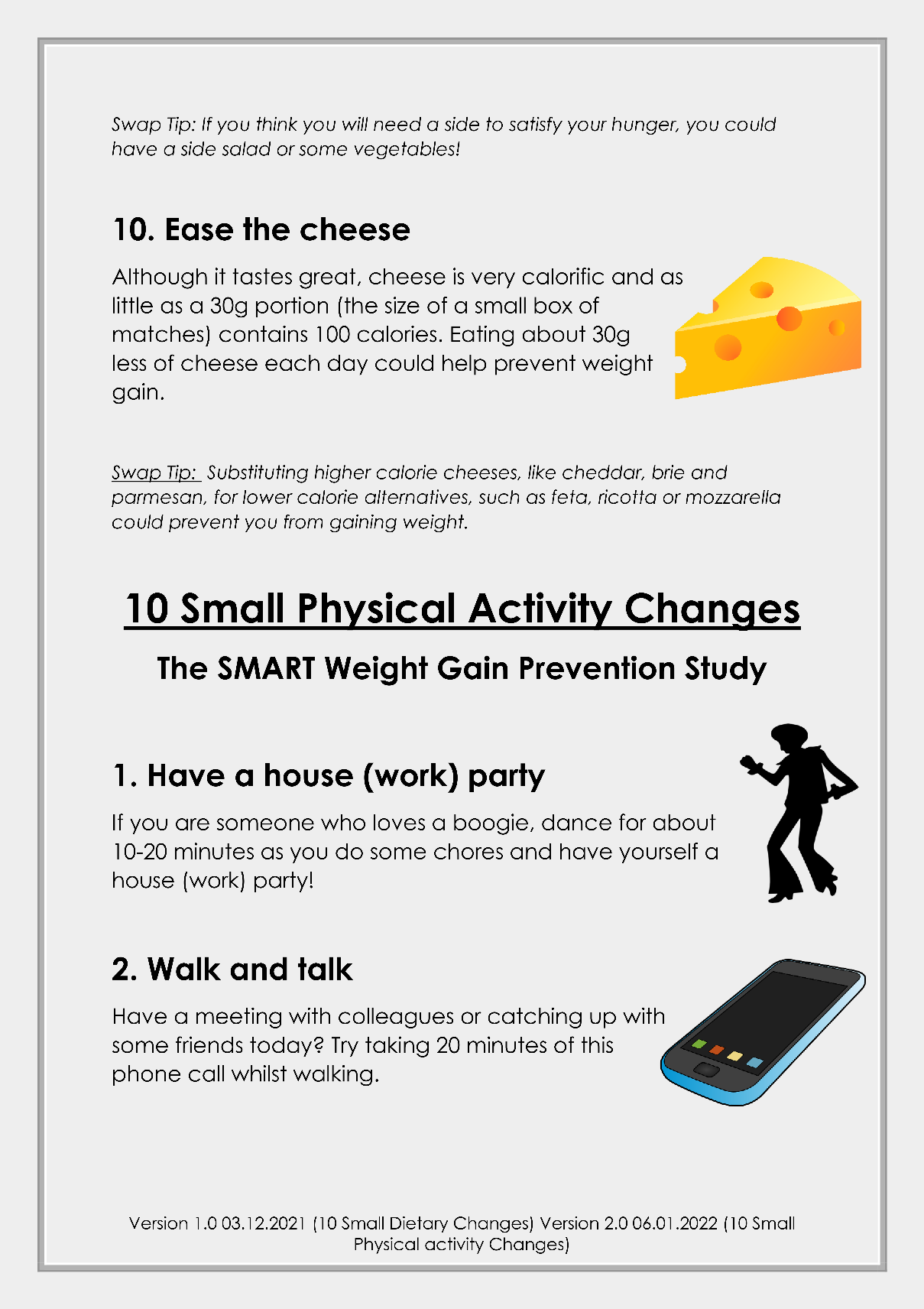


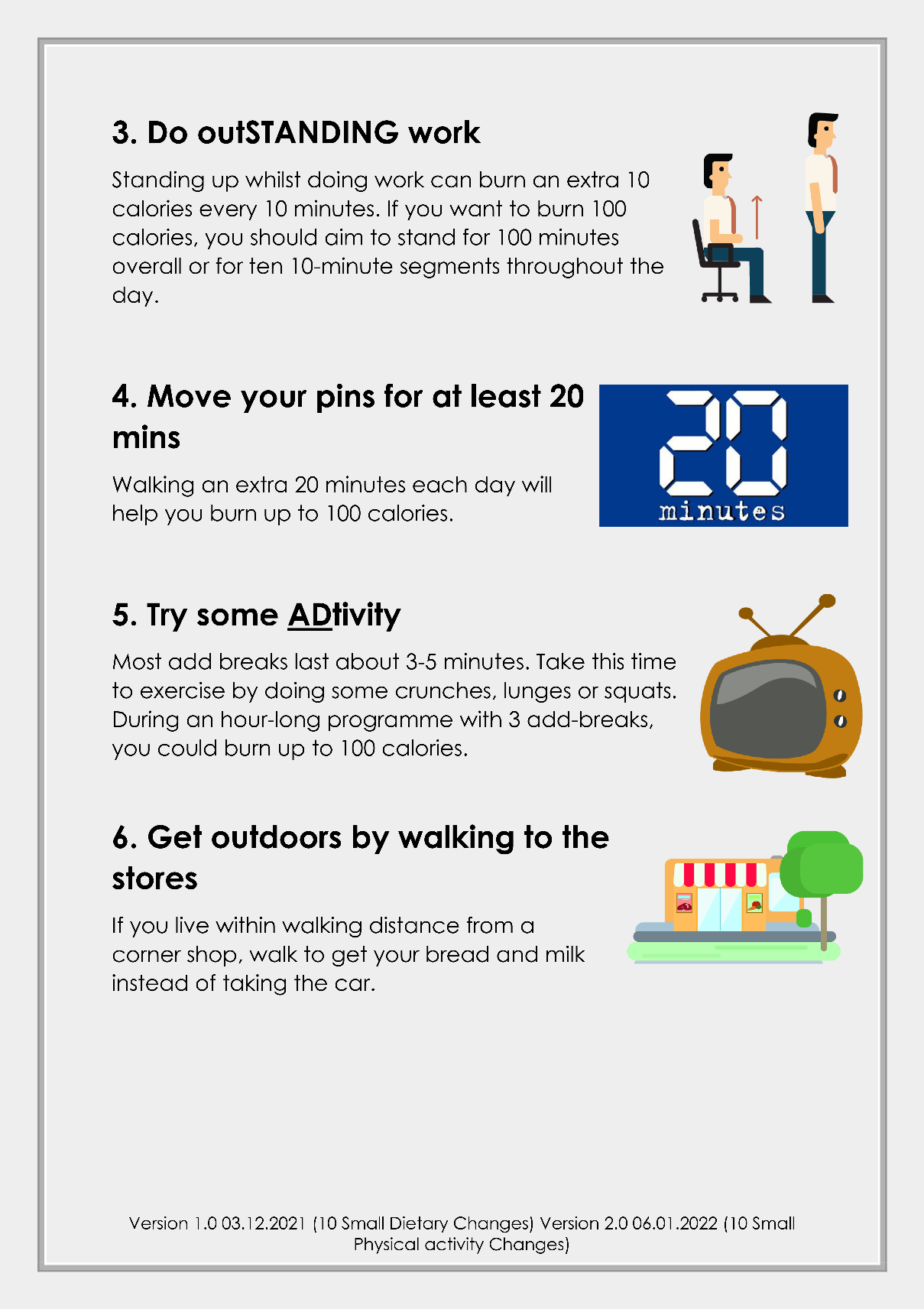


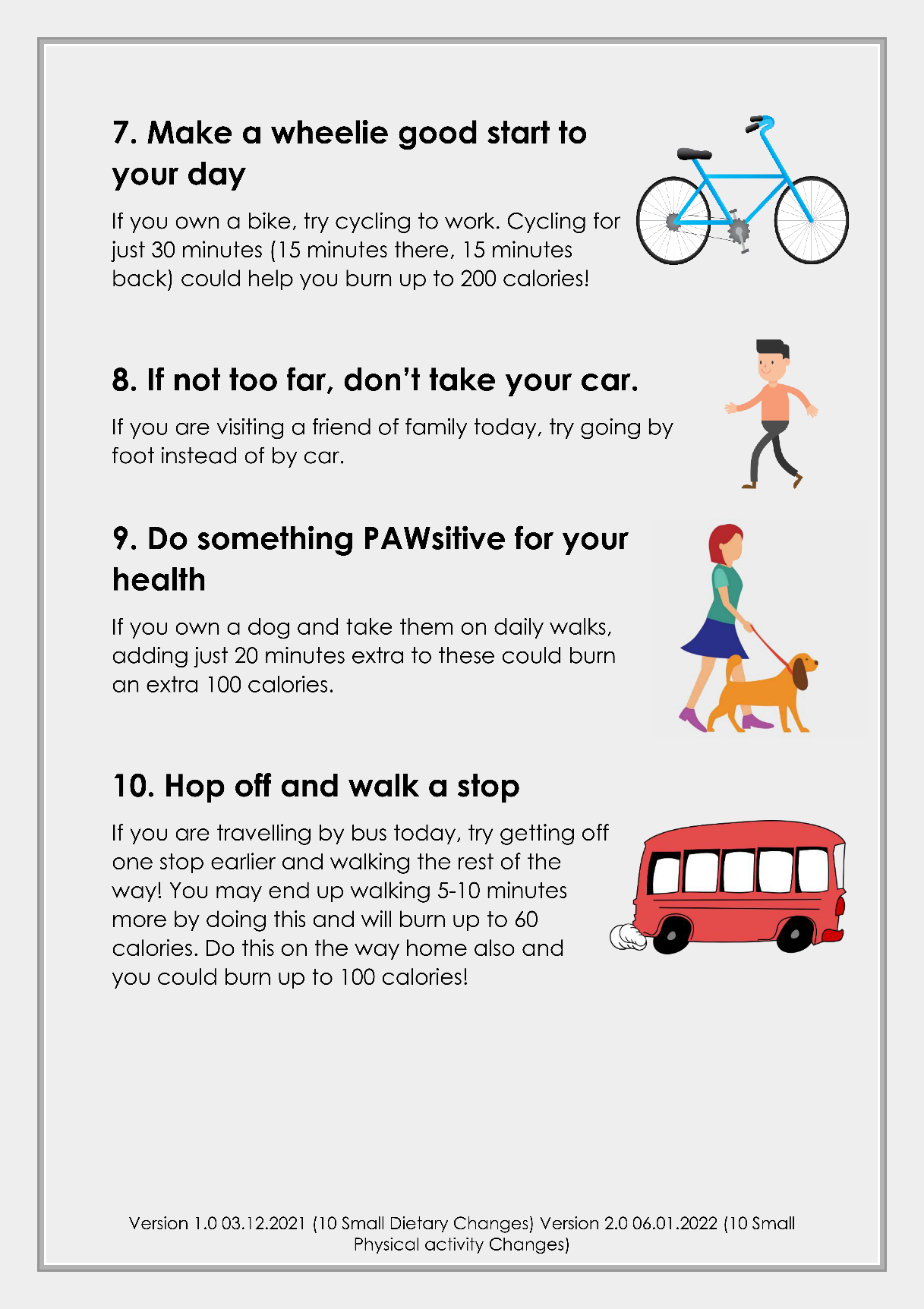


| **Table S1. Text messages sent throughout 12-week intervention.** |
| --- |
| **Goal Setting (1) x6** |
| Hello! Remember: your goal is to decrease the calories you eat and/or increase the calories you burn by 100-200 each day. We have some ideas of how to do this here (insert link). |
| It’s a new week! Remember to make one small diet and/or physical activity change a day. Choose one here (insert link)! |
| Remember-you don’t have to make the same small change every day, you can mix it up. Choose small changes that suit you (insert link)! |
| It’s Friday!! It’s hard to maintain routines at the weekends but small changes add up! You can do it! |
| Sometimes people lose motivation in the middle of the week. Try a different small change if you are bored of the others. See the list here (insert link) |
| If you can’t make one small change today, think about making two small changes tomorrow. |
| **Self-Monitoring and Review Behavioural Goal (2) x5** |
| To make sure you are reaching your goal, write down what small changes you make and the date you make them. You can use this diary if you wish (insert link). |
| Going into the weekend, don’t forget to write down the small changes you do and the date you make them. |
| Record all the small changes you make anywhere where it is easy for you- on your phone, post it note or in the diary we gave you (insert link) |
| Keeping track of the small changes you make will help you see if you are reaching your goals! |
| Recording the small changes you make and the date you make them, is one of the most important things you can do to successfully manage your weight. |
| **Discrepancy Between Current Behaviour and Goal & Problem-Solving (3) x6** |
| Will you have made at least 7 small changes by Sunday? |
| If you didn’t make at least 7 small changes last week, what might have got in the way? |
| If you’ve successfully made small changes this week, what’s helped you? Think about these as we head into the weekend! |
| What’s helped you make small changes? Is there any way that you could include these things into your life more to make it easier? |
| What’s stopped you from making small changes so far this week? Is there any way that you could exclude these from your everyday life to make it easier? |
| **Action Planning (4) x5** |
| Don’t forget, the best way to use a small change approach is to make one small change each day. Which small change will you make today? Write it down to remind yourself! |
| New week! Look at your schedule and think about what small changes will work for you this week! |
| Think about your week ahead. Can you make one small change each day? |
| Think about the week ahead. What small changes fit into your schedule? |
| One of the best tips to successfully make small changes is to plan ahead. |
| People who plan their small changes tend to be more successful in managing their weight. |
| **Bi-Weekly Questionnaires (6) x6** |
| We hope you have enjoyed making small changes to manage your weight this week! Please fill in this short (1 minute) questionnaire 😊 (insert link) |
| You are doing great! We are checking in today to see how many small changes you have been able to make this week-please fill in this short (1-minute) questionnaire (insert link). Thank you |
| You are halfway through the study Keep going, you can do it! Please fill in this short (1-minute) questionnaire (insert link). |
| We hope you have enjoyed making small changes this week. Please fill in this short (1-minute) questionnaire? (Insert link) |
| You only have two weeks left of this study! We hope you have found it useful so far. Please fill in this short (1-minute) questionnaire (insert link). |
| Thanks for you continued participation in our study. Please fill in this questionnaire (insert link). |
| **Other Messages (5) x5** |
| The difference between gaining weight or not gaining weight can depend on whether we eat just 100 extra calories (equivalent to about 2 custard cream biscuits)! |
| Adults tend to gain about 0.5 to 1kg (1-2 pounds) each year. This doesn’t sound like a lot but over time it all adds up! |
| Losing weight using the small change approach will take time. Try to stick with it 😊. Start by preventing weight gain first! |
| Managing our weight is hard, and if you’re struggling, you aren’t alone! Keep going. It will be worth it 😊 |
| Don’t feel unmotivated if your weight isn’t decreasing. Preventing weight gain is the first step in managing your weight! |

**Table S2. Timepoint of assessment for the primary, secondary and process outcomes**

| **Timepoint of assessment** | | | | | | | |
| --- | --- | --- | --- | --- | --- | --- | --- |
| Outcomes | Baseline | Week 2 | Week 4 | Week 6 | Week 8 | Week 10 | Week 12 |
| **Primary** | | | | | | | |
| Percentage of participants who made 7 small changes each week |  | X | X | X | X | X | X |
| Percentage of participants who found a small change approach helpful/very helpful |  |  |  |  |  |  | X |
| Percentage of participants who found the intervention materials helpful/very  helpful |  |  |  |  |  |  | X |
| Number of participants randomised per month |  |  |  |  |  |  | X |
| Retention of participants |  |  |  |  |  |  | X |
| **Secondary** | | | | | | | |
| Weight | X |  |  |  |  |  | X |
| MVPA  minutes/week | X |  |  |  |  |  | X |
| Dietary behaviour | X |  |  |  |  |  | X |
| **Process** | | | | | | | |
| Cognitive restraint of eating | X |  |  |  |  |  | X |
| Self-efficacy for dietary behaviours | X |  |  |  |  |  | X |
| Self-efficacy for physical activity behaviours | X |  |  |  |  |  | X |
| Self-regulation | X |  |  |  |  |  | X |

**Table S3. Number of small changes made across 12 weeks**

| Percentage of participants who reported they made 7 small changes within the bi-weekly  questionnaire | Week 2 N=76 | Week 4 N=63 | Week 6 N=60 | Week 8 N=55 | Week 10 N=58 | Week 12 N=55 |
| --- | --- | --- | --- | --- | --- | --- |
|  | 28 (n=21) | 25 (n=16) | 30 (n=18) | 22  (n=12) | 22 (n=13) | 18  (n=10) |
| Percentage of participants who reported they made 6 small changes within the bi-weekly questionnaire | 9  (n=7) | 16 (n=10) | 10 (n=6) | 11  (n=6) | 9  (n=5) | 9  (n=5) |
| Percentage of participants who reported they made 5 small changes within the  bi-weekly questionnaire | 12 (n=9) | 11  (n=7) | 15 (n=9) | 20  (n=11) | 12  (n=7) | 11  (n=6) |
| Percentage of participants who reported they made 4 small changes within the bi-weekly questionnaire | 8  (n=6) | 13  (n=8) | 8  (n=5) | 11  (n=6) | 10  (n=6) | 18  (n=10) |
| Percentage of participants who reported they made 3 small changes within the bi-weekly  questionnaire | 18 (n=14) | 10  (n=6) | 12  (n=7) | 16  (n=9) | 9  (n=5) | 11  (n=6) |
| Percentage of participants who reported they made 2 small changes within the bi-weekly questionnaire | 11 (n=8) | 13  (n=8) | 13 (n=8) | 5  (n=3) | 19 (n=11) | 20  (n=11) |
| Percentage of participants who reported they made 1 small changes within the bi-weekly questionnaire | 9  (n=7) | 6  (n=4) | 5  (n=3) | 5  (n=3) | 10  (n=6) | 5  (n=3) |
| Percentage of participants who reported they made 0 small changes within the bi-weekly  questionnaire | 5  (n=4) | 6  (n=4) | 7  (n=4) | 9  (n=5) | 9  (n=5) | 7  (n=4) |

**Table S4.** Examples of how participants implemented a small change approach

| **Examples** | **Number of participants who gave this**  **example** |
| --- | --- |
| Increased amount of physical activity | 29 |
| Resisted consuming certain indulgences | 8 |
| Reduced snacking between meals | 3 |
| Reduced portion size | 5 |
| Resisted consuming certain foods (not  indulgences) | 4 |
| Made food substitutions | 1 |
| Maintained current levels of physical  activity | 2 |
| Increased physical activity to compensate  for increased eating | 3 |
| Increased intensity of physical activity | 1 |
| Restricted time frame for eating | 1 |
| Reduced number of portions | 1 |
| Stopped buying indulgences | 1 |
| Started to track calories | 1 |
| Reduced indulgence intake | 3 |
